# Deep Brain Stimulation Initiative: Toward Innovative Technology, New Disease Indications, and Approaches to Current and Future Clinical Challenges in Neuromodulation Therapy

**DOI:** 10.3389/fneur.2020.597451

**Published:** 2021-01-28

**Authors:** Yanan Sui, Ye Tian, Wai Kin Daniel Ko, Zhiyan Wang, Fumin Jia, Andreas Horn, Dirk De Ridder, Ki Sueng Choi, Ausaf A. Bari, Shouyan Wang, Clement Hamani, Kenneth B. Baker, Andre G. Machado, Tipu Z. Aziz, Erich Talamoni Fonoff, Andrea A. Kühn, Hagai Bergman, Terence Sanger, Hesheng Liu, Suzanne N. Haber, Luming Li

**Affiliations:** ^1^National Engineering Laboratory for Neuromodulation, Tsinghua University, Beijing, China; ^2^Charité, Department of Neurology, Movement Disorders and Neuromodulation Unit, University Medicine Berlin, Berlin, Germany; ^3^Section of Neurosurgery, Department of Surgical Sciences, Dunedin School of Medicine, University of Otago, Dunedin, New Zealand; ^4^Department of Psychiatry and Behavioural Science, Emory University, Atlanta, GA, United States; ^5^Department of Radiology, Mount Sinai School of Medicine, New York, NY, United States; ^6^Department of Neurosurgery, Mount Sinai School of Medicine, New York, NY, United States; ^7^Department of Neurosurgery, University of California, Los Angeles, Los Angeles, CA, United States; ^8^Institute of Science and Technology for Brain-Inspired Intelligence, Fudan University, Shanghai, China; ^9^Harquail Centre for Neuromodulation, Sunnybrook Research Institute, Toronto, ON, Canada; ^10^Department of Neurosciences, Lerner Research Institute, Cleveland Clinic, Cleveland, OH, United States; ^11^Neurological Institute, Cleveland Clinic, Cleveland, OH, United States; ^12^Department of Neurosurgery, John Radcliffe Hospital, Nuffield Department of Surgical Sciences, University of Oxford, Oxford, United Kingdom; ^13^Department of Neurology, University of São Paulo Medical School, São Paulo, Brazil; ^14^Hospital Sírio-Libanês and Hospital Albert Einstein, São Paulo, Brazil; ^15^Department of Medical Neurobiology (Physiology), Institute of Medical Research–Israel-Canada (IMRIC), Faculty of Medicine, Jerusalem, Israel; ^16^The Edmond and Lily Safra Center for Brain Research (ELSC), The Hebrew University and Department of Neurosurgery, Hadassah Medical Center, Hebrew University, Jerusalem, Israel; ^17^University of Southern California, Children's Hospital Los Angeles, Los Angeles, CA, United States; ^18^Department of Neuroscience, College of Medicine, Medical University of South Carolina, Charleston, SC, United States; ^19^Department of Pharmacology and Physiology, University of Rochester School of Medicine & Dentistry, Rochester, NY, United States; ^20^McLean Hospital and Harvard Medical School, Belmont, MA, United States

**Keywords:** neuromoxdulation, depression, deep brain stimulation, MRI compatibility, gait disability

## Abstract

Deep brain stimulation (DBS) is one of the most important clinical therapies for neurological disorders. DBS also has great potential to become a great tool for clinical neuroscience research. Recently, the National Engineering Laboratory for Neuromodulation at Tsinghua University held an international Deep Brain Stimulation Initiative workshop to discuss the cutting-edge technological achievements and clinical applications of DBS. We specifically addressed new clinical approaches and challenges in DBS for movement disorders (Parkinson's disease and dystonia), clinical application toward neurorehabilitation for stroke, and the progress and challenges toward DBS for neuropsychiatric disorders. This review highlighted key developments in (1) neuroimaging, with advancements in 3-Tesla magnetic resonance imaging DBS compatibility for exploration of brain network mechanisms; (2) novel DBS recording capabilities for uncovering disease pathophysiology; and (3) overcoming global healthcare burdens with online-based DBS programming technology for connecting patient communities. The successful event marks a milestone for global collaborative opportunities in clinical development of neuromodulation to treat major neurological disorders.

## Introduction

The National Engineering Laboratory for Neuromodulation (NELN) at Tsinghua University organized its first deep brain stimulation (DBS) initiative meeting in Beijing on October 11–12, 2018. Leading experts in neuromodulation, specifically in the field of DBS, were in attendance for discussions on the latest research in neuromodulation technologies and applications, clinical indications, as well as current and foreseeable challenges in DBS therapy. Participants from multidisciplinary backgrounds that included neural engineers, neurosurgeons, neurologists, neuroscientists, and industry professionals engaged in round-table discussions following the thematic sessions and presentations. With expert updates and reports on the latest clinical approaches, there were open discussions on the opportunities in neuromodulation with recent technological advancements. This included an exchange of ideas on the connectome approach to DBS, novel developments of 3-Tesla magnetic resonance imaging (3T MRI)-compatible DBS devices and the use of neuroimaging to understand the neurocircuitry of effective DBS, including demonstrations of the latest DBS neural recording technology in real patients. This meeting came to provide reports of recent DBS application for unmet clinical needs, such as gait disability in Parkinson's disease (PD) and stroke rehabilitation, and the challenges in the current transition of DBS therapy toward neuropsychiatric disorders, including depression and memory disorders.

With the current rapid and widespread rise of neuromodulation therapies in China and across the globe, NELN's first DBS initiative meeting set out to stimulate collaborations between leaders in clinical, engineering, and basic science research for the rapid translation of therapies using state-of-the-art technologies. Marking a unique milestone in fostering international collaborations in DBS research, we report here a summary of the meeting that covers topic overviews, presentations, and follow-up discussions aiding to uncover expert perspectives and support advancements in the field of neuromodulation as we progress into the future.

## Some Recent Deep Brain Stimulation Technology Advancements

### MRI Compatibility

Among patients with an active implantable medical device, it is estimated that ~50–75% will require an MRI scan during the time course of treatment (1). In 2016, it was reported that 66–75% of DBS patients treated for movement disorders required an MRI scan within 10 years of device implantation (2). However, clear dangers exist for DBS patients under MRI, specifically the potential for permanent neurological damage due to radiofrequency (RF) lesioning caused by heating of DBS electrodes (3). Indeed, the main risk in MRI comes from wires enclosed in DBS extensions and leads, which can receive RF energy from the MRI magnetic field, inducing current discharge through contacts that align at the tip of the lead. This can cause thermal damage to the surrounding brain tissue. Therefore, inhibiting MRI RF-induced heat remains key for addressing the safety of patients implanted with DBS devices under MRI.

While some DBS device manufacturers claim safety under 1.5T MRI, DBS devices available to patients across global markets are still considered to be unsafe under 3T MRI, limiting patients from imaging and diagnostic benefits. Currently, patients can only use head/body coils for scanning under 1.5T, with the head specific absorption rate (SAR) value being <0.1 W/kg or the B1RMS value remaining below 2.0 uT (4). This is far lower than the upper limit of 2.0 W/kg of patients without medical devices for MRI (5), as set by the International Electrotechnical Commission (IEC) standard.

**Figure 1:**
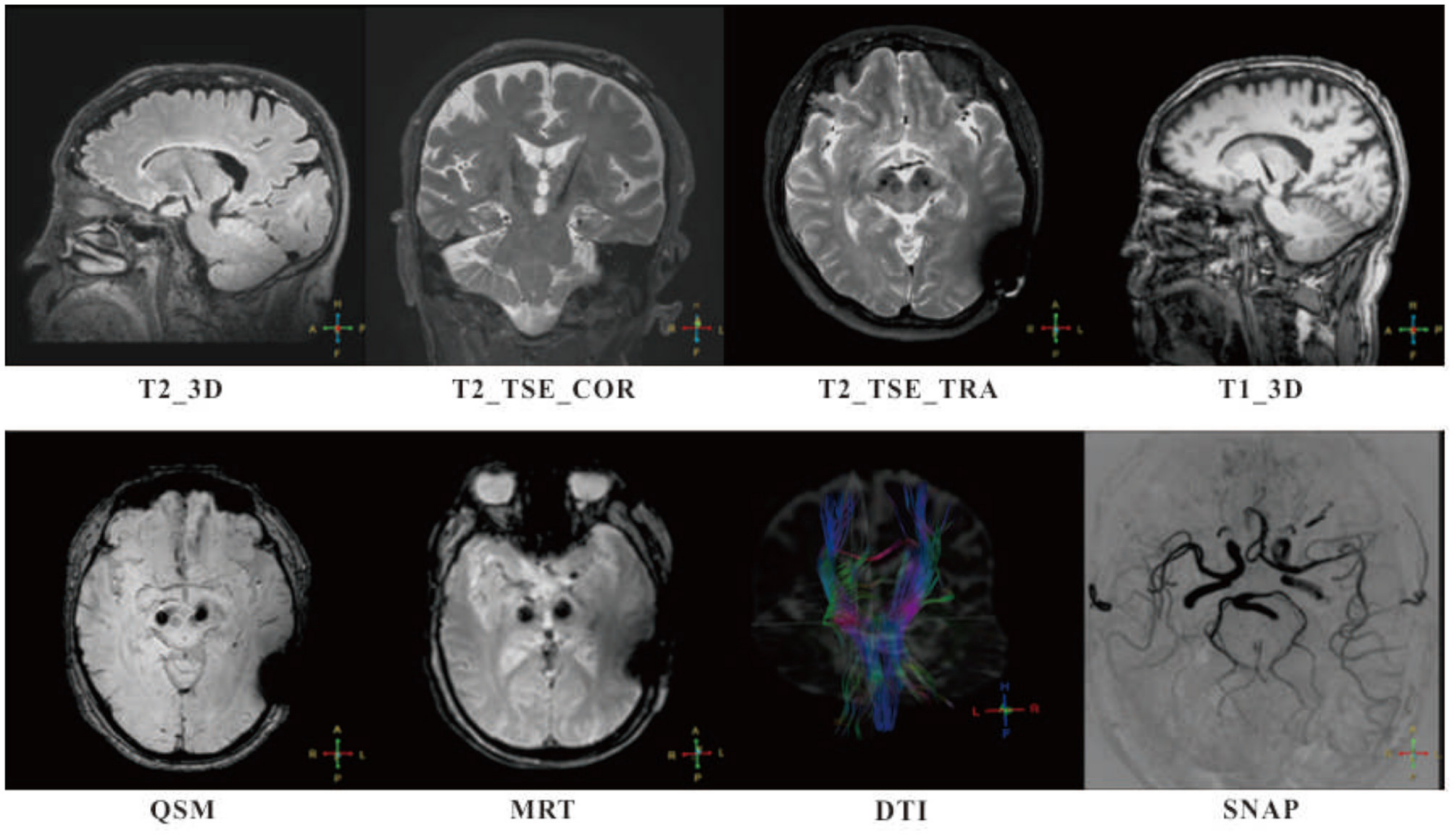
Follow-ups of patients with MR-compatible deep brain stimulation (DBS) implanted were successfully completed at 1, 3, 6, and 12 months, with intensive 3T MR scan. High in-plan resolution T2-weighted fast spin echo sequences (T2_TSE_COR and T2_TSE_TRA) and high specific absorption rate (SAR) isotropic sequences (T1_3D, T2_3D, and QSM) were adopted for anatomy analysis. Simultaneous non-contrast angiographies (SNAP) and diffusion tensor imaging (DTI) were taken for monitoring potential lesion on blood vessels and edema occurrence. A special sequence, magnetic resonance thermometry (MRT) used for tissue temperature online assessment. No adverse events were found that related to MRI.

With the limitations of low-quality imaging using 1.5T MRI, clinical research in DBS patients would greatly benefit from advances in MRI compatibility. Following laboratory evaluations and preclinical testing, a study conducted by the NELN was reported for testing the safety and efficacy of high-field 3T MRI-compatible DBS system in PD patients. The clinical trial was initiated in November 2016, and the final follow-up was completed in June 2018. A total of 24 PD patients were screened, and 14 subjects were eligible for the study. Follow-ups were successfully completed at 1, 3, 6, and 12 months, with an average time of 4.12 h taken per patient for anatomical and brain function 3T MRI scan. No adverse events were found that related to MRI (6) (Figure 1).

### Recording Neural Signals in Deep Brain Stimulation

Electrophysiological recordings of oscillatory neural networks remain an important tool for advancing brain research. In PD, β-band oscillations detected from the basal ganglia correspond to the degree of motor symptoms, such as rigidity and L-3,4-dihydroxyphenylalanine (L-DOPA)-induced dyskinesia, representing a pathophysiological marker of movement disorders that are beyond parkinsonism alone (7–10). Previous clinical studies have recorded neural activity intraoperatively during DBS surgery, with local field potentials (LFPs) being captured from external cables connected to DBS leads. With limited data recording and subsequent processing being completed after DBS implantation, along with potential complications from microlesion effect and edema along lead trajectories, studies of disease pathophysiology have been limited with such methods. Current implantable LFP-recording DBS systems are available but generally have a non-rechargeable battery lasting 3–5 years. Notably, these devices have high power consumption that results from LFP acquisition and readouts, limiting the time of use and requires earlier replacement of the implantable pulse generator (IPG), a factor that is not favored by patients. The need for extending the longevity of the DBS recording device remains at the forefront of neural engineering research. This would allow for efficient long-term LFP recordings, for example, to assess the changes of β-band oscillations in response to motor symptoms over time. Hopefully, more research on θ-band relating to tremor and prokinetic γ-band can help us develop robust algorithms for closed-loop control.

While the implantable DBS device PC+S produced by Medtronic has been used in previous research, its storage capacity in the IPG has limitations for long-term continuous recordings that often require massive LFP data storage. To resolve such issues, streaming of data to an external storage provides a solution for unlimited data collection and simultaneous assessment of physiological signals, such as movement behaviors, in a freely moving environment that allows for sophisticated clinical experimentation. The latest DBS devices for LFP recordings would be expected to include (1) large-capacity battery or wireless charging technology that fulfills long-term implantation acceptable to patients receiving treatment, (2) continuous high-precision data acquisition capability, (3) real-time external transmission that is wireless and therefore can be applied in freely moving conditions, and (4) multiple differential signal acquisition channels that can function simultaneously with DBS-ON, with sampling rates exceeding 500 Hz.

Here, the NELN demonstrated and reported PD patients implanted with DBS device G102RS from PINS Medical Ltd., a device engineered with rechargeable LFP-sensing and data streaming capacity. The first clinical trial has been completed in PD patients (*n* = 13) with a successful post-surgery follow-up through 12 months. Preliminary data have been used to characterize components of β-band oscillations during sleep states (11). Furthermore, it is reported that the response of β-band oscillations to high-frequency DBS is changed over time, which is a likely result from changes in neural network plasticity.

### Remote Online Deep Brain Stimulation Programming

Following implantation of a DBS device, postoperative DBS programming conducted by specialists is a vital part of achieving optimal clinical efficacy in patients (12). However, practical burdens in the clinical setting exist such as limited specialists available, time constraints, patient travel to specialist centers, and additional care costs (13). In recent years, the NELN has developed a remote online DBS programming system that operates with hardware-level protection for remote communication security (14). This has been specifically aimed at alleviating common healthcare burdens in the field of DBS worldwide. To date, the number of patients who have successfully used the remote DBS program control in China has exceeded 3,000 and has also been implemented between different countries, such as the UK, Spain, and Singapore, for patient management.

The development of the remote programming technology has notably reduced the burden of patient visits. In a recent survey with approximately 200 patients, costs and time-spent related to follow-up have both been reduced by >90%. Remote programming allows for clinical evaluations to be conducted through video and audio streams with Unified Parkinson's Disease Rating Scale (UPDRS)-III scoring and physical examination. As we look to the future, there is a natural evolution toward machine learning algorithm applications for automatic movement evaluation and objective output readings. Such applications allow for significantly increasing the amount of data collected on disease progression and enriching data pools for diagnoses and potential use toward future closed-loop systems.

### Variable Frequency Stimulation

High-frequency stimulation of the subthalamic nucleus (STN) through DBS in PD patients is a well-established application for alleviating parkinsonism. However, the application of high-frequency stimulation fails to alleviate axial disabilities in PD patients, which may occur due to disease progression, surgical injury, and side effects of electrical stimulation (15). Previous reports of low-frequency stimulation in PD have demonstrated the alleviation of axial disability but may compromise improvements in parkinsonism (16, 17). A recent pilot study completed has shown the promising effects of variable frequency stimulation (VFS), which applies alternating high- and low-frequency stimulations for freezing of gait (FOG) in PD patients (18). The long-term stability of VFS application is now being evaluated in a large clinical trial. In addition, video data collected from previous trials have been assessed with automatic classification and scoring based on machine learning methods to evaluate typical Timed Up and Go (TUG) tasks. This is now being utilized for objective classifications of FOG under separate analyses.

### Perspective of Artificial Intelligence for Deep Brain Stimulation

Artificial intelligence (AI) has great potential in medicine. Being broadly defined as the development of intelligent machines, the field of AI focuses on capabilities, such as understanding human languages and natural scenes, and development methods, such as machine learning (19–21). Machine learning entails building knowledge from patterns in data rather than being specified by human programmers. Much of the recent success in AI has come from the aggregation of massive training data and new computing systems for large-scale learning. New algorithms and systems have accelerated the widespread experimentation for prediction problem as supervised learning. In real-world situations, there is the desire to take strategies based on predictions. A next target for learning systems is data-driven decision-making.

**Figure 2:**
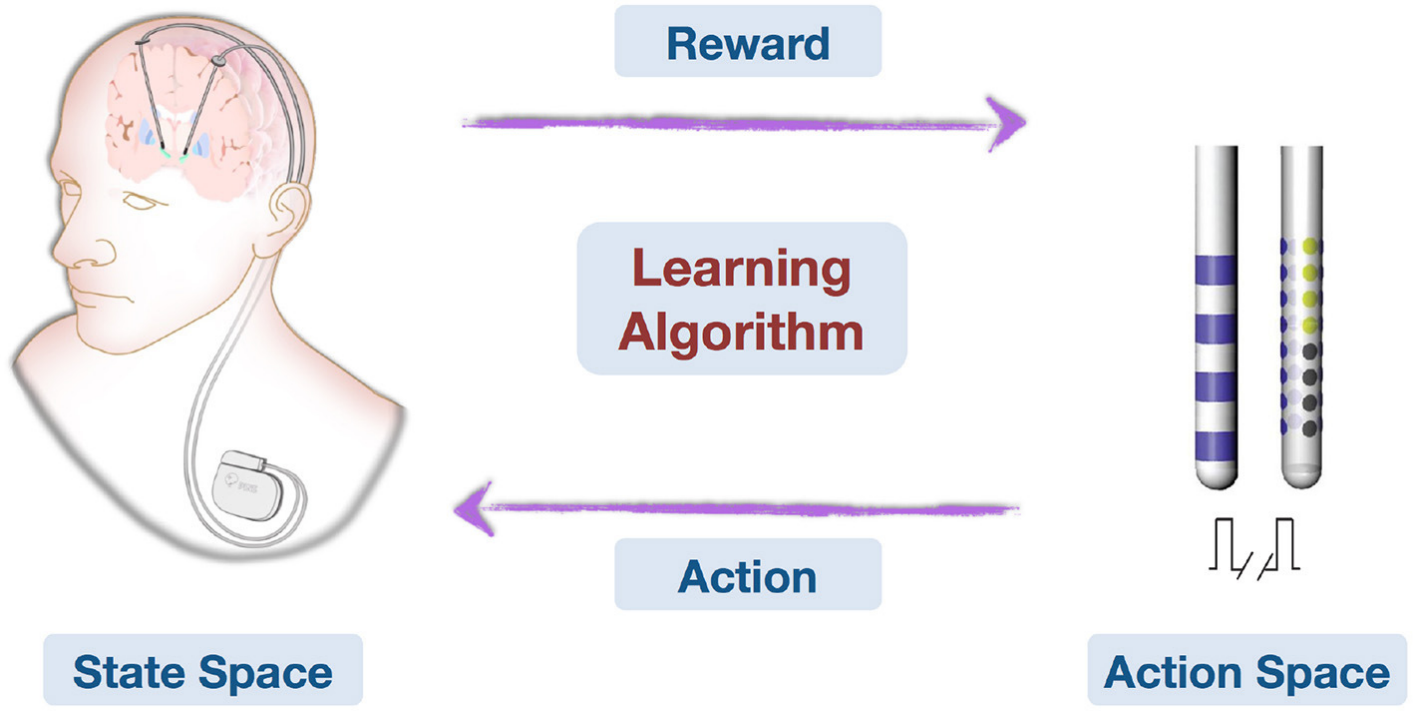
This is an illustration on how deep brain stimulation parameters can be learned and optimized online with feedback. Patient conditions are represented in the State Space. Action Space contains all possible stimulating patterns. The Learning Algorithm learns from domain knowledge and history data, then observes treatment effects (Reward) and optimizes stimulating pattern (Action) online.

Recent achievements in AI (including machine learning, computer vision, natural language processing) have the potential to improve our understanding of neurological disorders and corresponding treatments. In specific relation to DBS, there can be difficulty and time expenditure in finding optimal parameters for each patient. AI may help shape effective treatment for some of the most prevalent neurological disorders, such as PD, based on previous data (Figure 2). Future developments toward robust online learning techniques that explore large decision spaces and adapt to feedback in real time are essential to online learning problems. The application of AI techniques may allow us to uncover the mechanisms of DBS and the understanding of how DBS influences brain networks (11). It is noteworthy that recent advances in MRI-compatible DBS devices are allowing for acquisition of neuroimages during stimulation. It can be envisioned that a combination of advanced imaging techniques and AI techniques can facilitate the identification of DBS surgical targets in individual patients. Indeed, personalized implantation and automatic stimulation strategies can be aimed to maximize safety and efficacy of optimal treatment benefits and improve patient care.

Overall, advances in machine learning and robotics have the potential to improve health care delivery, from scheduling treatment plans to guiding surgical procedures, that are beyond current clinical capabilities. While these technologies shed light on the way toward better treatment, they also pose new challenges in terms of scale, complexity, safety, robustness, and efficacy.

## Innovations That Aim At Uncovering Deep Brain Stimulation Mechanisms of Action

### Toward Connectomic Deep Brain Stimulation

It was long thought that DBS exerts its function by local modulation of the target region itself, and a large number of studies have focused on such *local* effects by delineating optimal “sweet spots” for effective DBS. However, accumulating evidence suggests that DBS modulates fiber tracts or distributed brain networks and that such effects may be equally important for optimal treatment outcome (22–27). This has given way to a paradigm shift in the field of DBS, away from localized targeting and toward modulation of whole-brain networks by invasive neuromodulation.

In a parallel development, in the field of neuroimaging, the concept of the *connectome*, a formal mathematical description of brain regions and their interconnections was introduced in 2005 (28). Given the strong impact of the *connectomics* concept on the neuroimaging field, it is somewhat surprising that, so far, only a handful of studies have applied it to DBS (22, 29–31). One reason for this may be that patient-specific connectivity data [resting-state functional MRI (rs-fMRI) or diffusion MRI (dMRI)] are usually not acquired within clinical routine and are hard, if not impossible, to acquire postoperatively.

**Figure 3:**
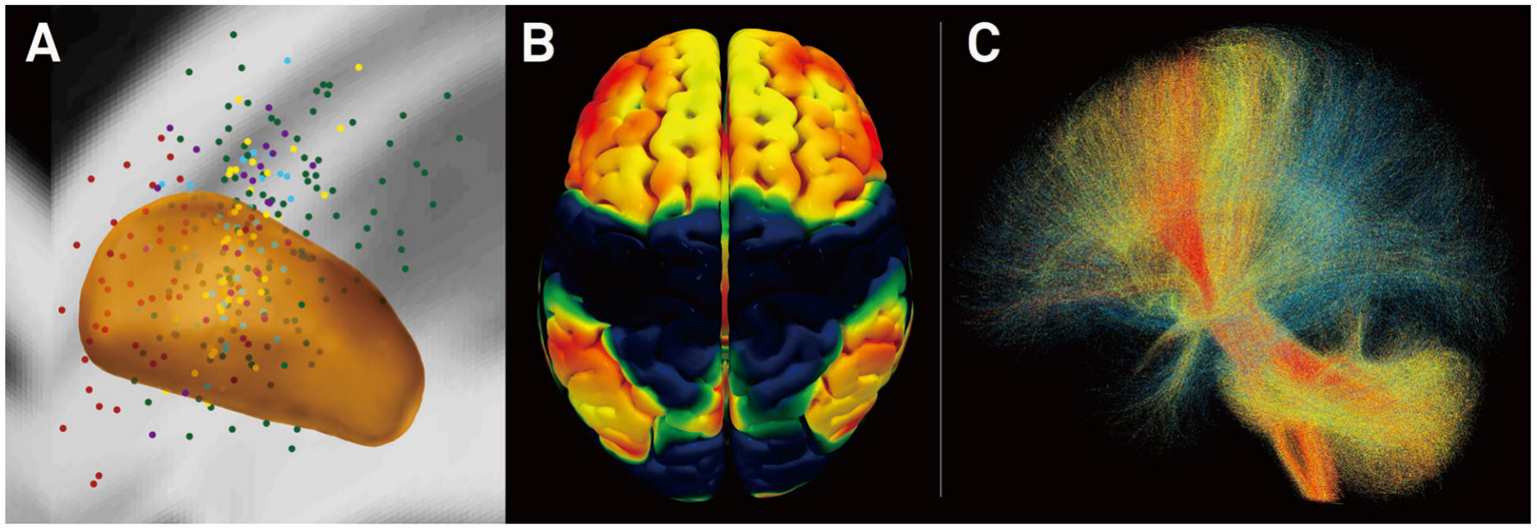
Connectivity predicts deep brain stimulation (DBS) outcome in Parkinson's disease (25). **(A)** Active stimulation coordinates from five cohorts out of two DBS centers mapped to subcortical anatomy [subthalamic nucleus (STN) shown in orange]. **(B)** Cortical connectivity map predictive of clinical outcome analyzed using normative-connectome based on resting-state functional MRI (rs-fMRI). Hot colors show areas that are associated with good clinical outcome if the electrode is strongly connected to them. In contrast, functional anticorrelation to areas in cold colors is associated with beneficial outcome. **(C)** Fiber tracts associated with good (red), intermediate (yellow), or poor (blue) clinical outcome. Connectivity profiles shown in **(B,C)** are able to predict motor improvement in out-of-sample data (across DBS cohorts and centers; R in the range of 0.5; ~20% variance explained).

To overcome this limitation, Horn et al. (23, 32–34) established a method that combines normative connectomes, i.e., average brain connectomes that are estimated on large cohorts of subjects, with DBS electrode reconstructions from a single patient. This concept has been successfully applied to other areas of clinical neuroimaging, for instance, to map stroke symptoms to brain regions (26, 28, 35) or to explain varying results of transcranial magnetic stimulation (TMS) treatment (36). A recent publication has demonstrated the feasibility of this concept for DBS (23). Here, the authors estimated the structural and functional connectivity profile of effective ventral intermediate (VIM) nucleus DBS by transforming an optimal literature-based DBS coordinate to standard stereotactic [Montreal Neurosciences Institute (MNI)] space and combining it with normative connectomes. A second study demonstrated that clinical DBS improvement can be predicted based on the connectivity profiles of electrodes alone (25) (Figure 3). Specifically, the structural and functional “connectivity fingerprints” of DBS electrodes in 95 PD patients operated on at two centers were highly predictive of their clinical motor improvement. In fact, the optimal connectivity profile of effective STN-DBS could be informed exclusively on data from the first DBS center and then used to accurately predict outcome in patients from the second center. The study demonstrated that brain connectivity may play a crucial role in the DBS mechanism of action and that it may be used to predict treatment outcome across cohorts and centers. Recently, the concept was transferred to essential tremor (37) and obsessive–compulsive disorder (OCD) (38). After further validation, resulting “effective treatment networks” of these and similar studies could in the future be used to guide both DBS programming and surgery. Moreover, networks could potentially be used to guide non-invasive brain stimulation since they define cortical areas that may play a role in disease-specific and therapeutic circuitries.

### Studying Effects of Deep Brain Stimulation in Individual Patients

DBS is a well-established functional neurosurgical technique that has recently observed rapid development as a potential treatment for neuropsychiatric disorders (27, 39). Not only does DBS mimic the effects of neuropharmacological treatment, but it currently offers key advantages with fewer side effects and greater adjustability. Although DBS has achieved great success in treating movement disorders such as PD and dystonia, a broader use of DBS for other neurological disorders is facing two major challenges. The first lies in accurate application in individual patients, as patients with neurological and psychiatric disorders are highly heterogeneous in terms of their symptom expression, disease progression, and more importantly their brain functional network organization. A personalized implantation/stimulation strategy is thus necessary to maximize the treatment benefits and improve patient care. The second challenge is the lack of in-depth understanding of how DBS impacts wider brain networks largely due to the lack of means to study the immediate and long-term stimulation effects on large-scale brain networks *in vivo*. A better appreciation of the mechanism of DBS is crucial in order to extend this important technique from treating movement disorders to a broader spectrum of brain diseases including Alzheimer's disease (AD), stroke, and neuropsychiatric disorders.

Understanding the neurophysiology, connectivity, and neuropathology at the level of individual patients is key to furthering the success of DBS treatment. To date, DBS applications are largely based on the presumption that current models of disease, which are predominantly derived from neuroimaging studies that identify brain abnormalities at a group level (40), can be directly applied to individual patients. However, it is becoming increasingly recognized that interindividual variability exists not only in macroscopic and microscopic brain anatomy (41–43) but also in the organization of functional systems, i.e., the topography and connectivity of functional regions may vary drastically across individuals (44, 45). Compared to unimodal sensory and motor functions, higher-order cognitive functions demonstrate substantial variability across individuals. Recent studies suggest that the high level of interindividual variability in higher-order functions may be a fundamental principle of brain organization and a critical outcome of human brain evolution (44–46). Disease models concerning motor circuits, which have a relatively low degree of interindividual variability, might be directly applied to individual patients to guide DBS treatment. For example, targeting the STN and globus pallidus internal segment (GPi) provides efficient treatment of akinesia, tremor, and rigidity in most PD patients (47). However, even with well-defined targets, not all patients seem to benefit from DBS to the same degree. The picture becomes more complicated with the application of DBS in psychiatric disorders. For example, a recent report of the application of DBS to the subcallosal cingulate for treatment-resistant depression yielded unsatisfactory results, with DBS failing to demonstrate a superior effect to sham stimulation (40). A subsequent trial has suggested that DBS targeting and parameters need to be optimized for individual patients in order to demonstrate treatment efficacy (48). Specifically, it was found that treatment responders shared a common pattern of white matter connectivity within the subcallosal cingulate region (49). These results suggest that it is necessary to develop patient-specific targets and cortical responsivity measures to identify precise DBS targets. On a systems level, it is crucial to develop non-invasive metrics of brain functional and structural connectivity, at an individual level, to make DBS treatment viable and to improve the cost–benefit ratio for patients. Recent technical advancement in functional connectivity MRI research has made it possible to localize functional networks at the single-subject level (50–52), which could thus be used to guide personalized DBS treatment. For example, the work by Wang et al. (52) has established a technology to parcellate cortical functional networks in individuals, which is highly sensitive to the characteristics of the individual and is able to capture intersubject variability. Functional networks localized using this parcellation technology were also validated by invasive cortical stimulation mapping in surgical patients. Such techniques may be essential for identifying DBS targets in individual patients in the near future.

**Figure 4:**
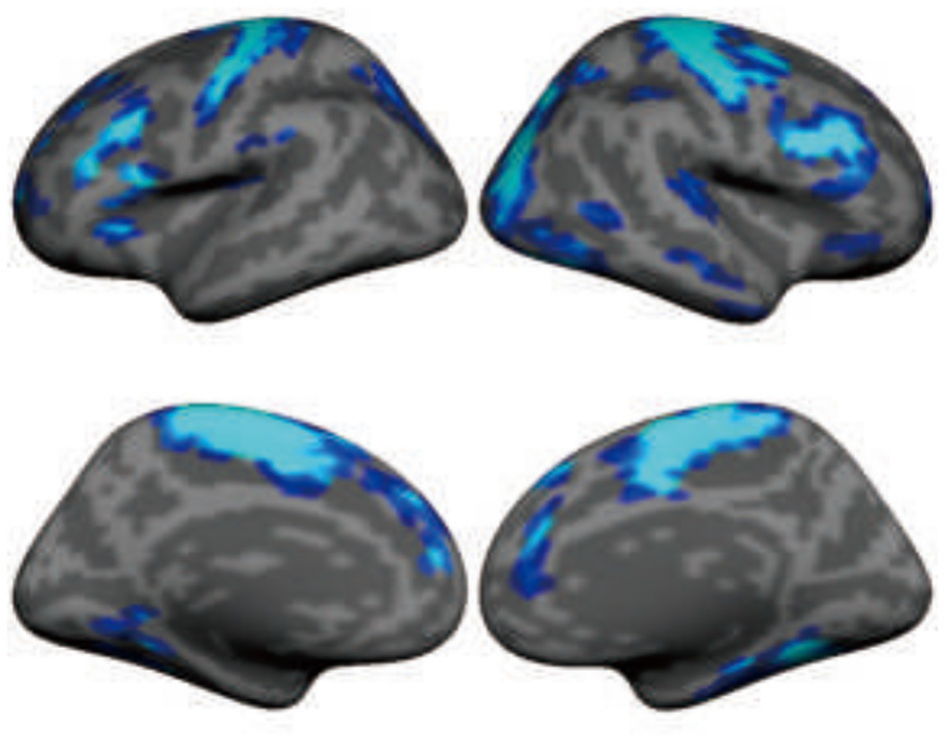
Subthalamic nucleus stimulation suppresses functional activity in large-scale brain networks, including sensorimotor and association regions in the frontal lobe. The map shows the functional MRI (fMRI) contrast between “DBS on” condition and “DBS off” condition in 11 patients with Parkinson's disease. Functional data were recorded using 3-Tesla MRI when deep brain stimulation (DBS) was turned on (36-s blocks) and off (24-s blocks) using a block design.

Understanding the immediate and long-term effects of DBS on large-scale brain networks requires technologies that can read out brain signals *in vivo*. Until recently, due to technical constraints, the local and remote effects of DBS have only been measured with electroencephalography using external leads and formed the basis for the investigation of brain response to DBS (53). Recent technological developments allow for the concurrent recording of LFPs and high-field MRI during DBS (54). The implications of these developments are profound (47). DBS significantly suppressed beta activity (13~35 Hz), but the suppression effect appeared to gradually attenuate during a 6-month follow-up period after surgery (55). The concurrent recording of LFPs allows for the characterization of pathophysiological neuronal firing patterns, the investigation of the clinical response according to application parameters, and the development and testing of new disease models. This technological advancement has benefited the study of, for example, the abnormal oscillatory activity (13–35 Hz) in PD, the pivotal role of the STN in basal ganglia physiology and pathophysiology, and the use of β-band oscillations as biomarkers to devise closed-loop DBS systems to deliver a more neurophysiologically efficient therapy. Nevertheless, electrophysiological signals recorded from implanted electrodes only reflect neural responses at local structures rather than the effects on large-scale, distributed functional networks. Obtaining a comprehensive picture of DBS effects on the human brain is now possible, thanks to the recent development of DBS devices that are compatible with high-field MRI (54). Taking advantage of these novel devices, we are able to record functional activity across the entire brain during DBS using 3T MRI. The high-quality imaging data can capture the changes in the large-scale brain networks when DBS is turned on and turned off. The data presented by Liu et al. (52) demonstrate immediate, strong suppression of brain activity in the sensorimotor cortex after STN stimulation in PD patients (Figure 4). This DBS-fMRI technology allows for examining the validity of potential DBS targets for a variety of brain disorders, eventually leading to a broader use of DBS.

Taken together, DBS has the potential to revolutionize the treatment of neurological and psychiatric disorders and to improve our understanding of human brain function. However, before DBS can be implemented into standard practice for a broad range of disorders, a better understanding of how it affects large-scale brain networks and identification of precise targets in individual patients are necessary. The development of individualized functional imaging techniques and MRI-compatible DBS devices will greatly facilitate research in this important field.

### Neurocircuitry Underlying Effective Deep Brain Stimulation for Mental Health Disorders

DBS is a promising therapeutic approach for patients with treatment-resistant mental health disease, including OCD and major depressive disorder (MDD) (56–58). MDD and OCD involve key elements of the cortico-cortical and cortico-basal ganglia networks. These networks include the ventromedial prefrontal cortex (vmPFC), orbitofrontal cortex (OFC), dorsal anterior cingulate cortex (dACC), and the basal ganglia structures, striatum, and STN (59). DBS primarily targets myelinated fibers that carry information from and to the above structures. As such, the most common DBS targets are (1) the subgenual cingulate gyrus white matter (SCGwm), the white matter adjacent to cortical areas 32 and 25; (2) the anterior limb of the internal capsule (ALIC) that carries descending and ascending cortical fibers; and (3) the connections of the STN, including the hyperdirect pathway that carries cortico- STN fibers (56–58).

The work by Haber et al. (59) has used a combination of nonhuman primate (NHP) tracing experiments and NHP and human dMRI to delineate the organization of PFC fiber pathways, which allows insight into which connections are likely to be involved at each DBS electrode site. The work has focused on how cortical fibers are organized within the SCGwm, ALIC, and STN and the fibers and terminal fields likely to be affected by DBS electrodes placed within those regions.

**Figure 5:**
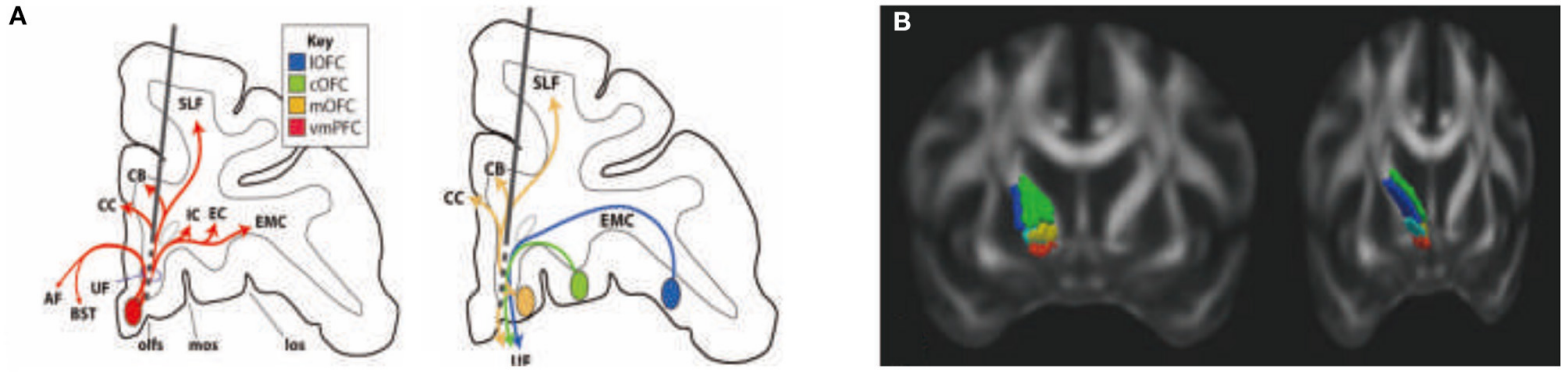
Rules ventral prefrontal cortical axons use to reach their targets: implications for diffusion tensor imaging tractography and deep brain stimulation for psychiatric illness (61). Organization of fibers passing through the subgenual cingulate gyrus white matter (SCGwm) **(A)** and anterior limb of the internal capsule (ALIC) **(B)**. **(A)** Schematic of an electrode passing through the SCGwm, indicating the cortical fibers involved at each contact. AF, amygdala fugal pathway; CB, cingulate bundle; CC, corpus callosum, EC, external capsule; EMC, extreme capsule; IC, internal capsule; los, lateral orbital sulcus; mos, medial orbital sulcus; olfs, olfactory sulcus; SLF, superior longitudinal fasciculus; UF, uncinate fasciculus. **(B)** Organization of fibers in the human ALIC. Red, OFC and vmPFC fibers; yellow, ventrolateral PFC fibers; light blue, dACC fibers; green, dorsolateral PFC fibers; blue, dorsomedial PFC fibers.

The SCGwm site is primarily used for treatment-resistant depression. The most effective SCGwm contacts (1 and 2) are at the border between the SCG and the inferior rostral gyrus (60). Fibers that pass through this region include multiple connection involving the entire ventral surface of the frontal cortex (Figure 5A). Contact 1 is within the inferior rostral gyrus white matter, contact 2 is within the SCG, and contracts 0 and 3 are ventral and dorsal, respectively. Contacts 0–2 will involve (1) all connections from vmPFC areas adjacent to the electrode contacts (both cortical and subcortical projections); (2) uncinate fasciculus fibers from non-adjacent vmPFC and medial OFC as they travel medially to other ventral PFC areas; (3) a subset of lateral OFC fibers traveling medially to innervate medial PFC areas; (4) axons traveling from the contralateral vmPFC and medial OFC; and (5) a subset of anterior vmPFC and medial OFC en route to the corpus callosum through the uncinate fasciculus. Contact 3 involves primarily fibers in the corpus callosum. In addition, this site captures a subset of fibers traveling from the medial OFC and posterior lateral OFC to the cingulum bundle and superior longitudinal fasciculus (61).

The ALIC site is used for both treatment-resistant MDD and OCD. Fibers from different cortical regions follow predictable trajectories to, and locations within, the ALIC. The relative position of fibers from different cortical areas, within the ALIC, demonstrates the topology, and specifically the ALIC segmentation, based on PFC origin of fibers. Fibers arising from dorsal regions travel within the capsule dorsal to those from ventral cortical areas. Axons derived from medial areas travel within the capsule medial to those from lateral regions (Figure 5B). The organization shows how stimulation in different locations throughout the ALIC is likely to impact projections of different cortical areas, including the ventrolateral PFC (vlPFC), dorsolateral PFC (dlPFC), dorsomedial PFC, and dACC. Each contact placed within the ALIC activates a different subset of corticothalamic and brain stem fibers. In particular, an electrode contact targeting the ventralmost part of the ALIC will likely impact primarily fibers from the vmPFC and OFC. More dorsal contacts/lesions will impact lateral OFC, ventrolateral PFC, and dACC fibers. The most dorsal contacts/lesions will impact primarily the dorsomedial and dorsolateral PFC. In addition to consideration of the dorsoventral position of electrodes or lesions, the rostrocaudal position is also important (61, 62).

The effectiveness of DBS for depression at the SCGwm and ALIC sites has not been directly compared with respect to patient selection criteria. Nonetheless, both sites are effective in over 50% of otherwise treatment-resistant patients (63, 64). Stimulation at the SCGwm site captures all cortical and subcortical projections from the area surrounding each contact site. However, it also captures fibers from non-adjacent cortical areas passing through the target, including connections between different vPFC areas, and OFC fibers traveling to corpus callosum, medial forebrain bundle (MFB), cingulum bundle, and superior longitudinal fasciculus. In addition, this target captures the extensive brain stem connections from the SCG. In contrast, ALIC site does not directly involve corticocortical fibers. Rather, each contact in the ALIC site involves a different combination of thalamic and/or brain stem bundles. Of particular importance is that both DBS targets capture subsets of fibers that include both thalamic and brain stem fibers. Thus, an important part of the clinical effectiveness of DBS is likely to require a combination of thalamic and brain stem fibers.

The STN site, commonly used for PD is now experimentally used for OCD. In addition to the STN connections to both pallidal segments, there is an important direct cortico-STN connection that is referred to as the hyperdirect pathway. This pathway is also organized in a specific and general topographic manner. M1 projects to the dorsolateral STN, with area 6 projecting ventromedially to these terminals. Overall, PFC projections are concentrated and anterior to motor control projections. While dorsal PFC projections occupy the medial half of the STN, vmPFC and dACC dense terminal fields are located in the rostral and anterior, medial tip. This area also receives the limbic input from the pallidum, in particular, projections from the ventral pallidum (65). The delineation of limbic and cognitive hyperdirect pathways has been key for developing a DBS site for the treatment of OCD (58). It has also contributed to our understanding of non-motor effects of DBS in PD. Taken together, DBS for motor disorders such as PD targets the lateral STN regions, while the target for OCD targets the medial STN. Effectiveness of DBS for OCD at the ALIC and STN sites is now being compared at several clinical sites.

## Deep Brain Stimulation for Major Depression and Addiction

MDD has been known to exist since the origins of humankind. Hippocrates (460–370 BC) referred to MDD as “melancholy.” Being a highly heterogeneous disorder, symptoms of MDD affect a range of behavioral domains including mood, sleep, sexual behavior, and motor functioning. MDD has a 1-year prevalence of 3–5% and lifetime prevalence of 15–20%. It has been reported that MDD is on the uprise (increase of 10% between 2005 and 2010), leading to an incremental economic burden for individuals with MDD. The cost increased by 21.5% (from $173.2 billion to $210.5 billion) (66).

DBS for MDD has demonstrated clinical benefit in three brain regions in open-label trials, including the ventral capsule/ventral striatum (VC/VS), the subgenual cingulate cortex (SCC), and the superolateral branch of the medial forebrain bundle (sl-MFB) (40, 67, 68). Two large, industry-sponsored sham-controlled randomized controlled trials (RCTs) of DBS for depression failed, one targeting BA25 and one targeting the ventral capsula (68). The BA25 trial (BROADEN) was halted when interim analyses showed a low likelihood of meeting primary endpoints. The outcomes of these trials have caused the field to question fundamental aspects of these therapies, including patient selection, trial design, network targeting, funding, and, of course, efficacy itself.

A number of lessons can be gleaned from these trials. (1) The underlying disorder, major depression, is not well-understood. We need better biomarkers to further delineate and stratify various forms of depression. (2) There is a need for identification of better outcome measures analogous to those used in DBS for movement disorders. For example, specific motor variables such as bradykinesia, rigidity, and tremor are utilized to determine the outcome of DBS for PD while psychiatric scales tend to focus on more holistic and subjective disease outcomes. Furthermore, these variables could also be used to identify patients most likely to respond to DBS. Thus, the best PD candidate is not simply the patient with the worst overall disease but one with specific levodopa-responsive symptoms. (3) There is shift, now, from the single-target approach to a network-based model of neuropsychiatry in which target selection is patient and disease specific. (4) We need to leverage structural and functional neuroimaging to better identify these network nodes. (5) We need to change our attitudes with regard to trial designs to support more flexible designs and to aggregate data across trials. (6) Finally, ongoing advancements in DBS hardware and software (such as directional electrodes and closed-loop devices) will result in increasing the therapeutic index and efficacy of DBS for psychiatric disorders. Thus, the field of psychiatric neurosurgery finds itself at a crossroads. Despite the setbacks of these so-called “failed” trials, the clinical and financial burden of psychiatric diseases continues to grow, as does the theoretical rationale for DBS therapy, with increased commitment from various national and international agencies.

In addition to major depression, substance addiction is one of the most prevalent and costly health problems globally. Standard medical therapy is often not curative, and relapse is common. Research over the past several decades on the neural underpinnings of addiction has implicated a network of structures within the brain shown to be altered in patients with substance abuse. While invasive neuromodulation such as DBS and VNS have proven to be effective in treating depression, OCD, and epilepsy, there is increasing interest and data with regard to their potential application in the treatment of severe, intractable substance abuse and addiction. Several neuromodulatory techniques and brain targets are currently under investigation in patients with various substance abuse disorders (69).

The current work by Bari et al. (70, 71) is aimed to apply the lessons learned from DBS for depression toward the application of DBS and other forms of invasive neuromodulation toward addiction. Thus, using probabilistic tractography, Bari et al. have identified specific limbic structures associated with nicotine addiction and impulsivity (currently under review with human brain mapping). In addition, brain mapping data from patients undergoing DBS for post-traumatic stress disorder (PTSD) combined with a normative connectomic approach has supported the role of the amygdala in regulating reward-related emotions. These efforts provide the background on which to design more informed trials of invasive neurmodulation for nicotine and other forms of addiction.

### Toward Deep Brain Stimulation Targets and Stimulation Designs for Depression

The neural correlates of MDD have only been partially unraveled and involve both activity and connectivity changes. Based on neuroimaging, a neural circuit taxonomy for depression and anxiety has been developed (72). This suggests that rumination is the consequence of hyperconnectivity within the default mode network, inattention due to hypoconnectivity within the frontoparietal attentional networks, anhedonia and context insensitivity to a dysfunctional positive affect network, and anxious avoidance to hypoconnectivity within the salience network and hyperconnectivity between salience and default mode network (72).

Before medications were discovered that could treat MDD, psychosurgical techniques were developed to address this complex pathology. Whereas, the initial approach was to perform a large frontal lobotomy, the development of stereotactic approaches in 1947 permitted smaller and better targeted lesions resulting in four kinds of psychosurgery: (1) cingulotomy, (2) anterior capsulotomy, (3) subcaudate tractotomy, and (4) limbic leucotomy (combination of 1+3). Even though psychosurgery came under public attack and was nearly forbidden, a dilemma arises with recent disinterest and disengagement of the big pharma in developing novel medications for brain disorders.

Based on modern structural imaging with tractography, it is now clear that these four targeted regions functionally converge at the pgACC, extending into the OFC, and are connected *via* the forceps minor and the anterior thalamic radiations to subgenual cingulate regions. Anatomically, this convergence may derive from the superolateral branch of the MFB, a structure that connects these frontal areas to the origin of the mesolimbic dopaminergic “reward” system in the midbrain ventral tegmental area, which is a possible final common pathway.

From the initial work by Mayberg et al. (49) the subgenual anterior cingulate has been selected as a target for depression, yet others have targeted the MFB. However, there seems to be a problem. Open-label studies for MDD are all positive including a meta-review of meta-analyses. Yet two controlled trials for MDD were both negative, one targeting BA25 and one targeting ventral capsule/striatum. This is in keeping with a larger problem that today, no target—whatever the disease—can meet the criteria for clinical efficacy as recently defined by an international committee for neurosurgery for psychiatric disorders. How can we overcome this problem? Should the neuromodulation community look for new targets, use novel stimulation designs, or a combination of the two?

Based on historical data from destructive psychosurgery, as well as modern functional and structural imaging, new targets for neuromodulation can be proposed: the dACC has been a target for the treatment of MDD with lesioning, TMS, and implants. Similarly, functional imaging suggests that also the left amygdala, right parahippocampal area, pgACC, caudate nucleus, insula, as well as the DLPFC and VLPFC could be potential targets for neuromodulation for MDD.

Another critical question is what is the ideal pattern of stimulation? DBS for movement disorders applies fairly standardized stimulation parameters, consisting of a frequency of 130 Hz, pulse widths <300 μs and variable amplitudes. Recently, burst stimulation has been developed, and this stimulation design applies the natural frequency of the targeted area, e.g., 6 Hz at the ACC, 20 Hz at the DLPFC, 4–8 Hz at the somatosensory and auditory cortex. Thus, effective outcomes can only be expected if both the target and stimulation design match. Interestingly, novel stimulation designs such as noise stimulation can be developed to prevent the brain of habituating to the stimulation. Noise can come in various forms, also called colors, from white to pink to brown and black, with an increasing steeper slope, following a 1/f^β^ with β = 0, 1, 2, 3 for white, pink, brown, and black, respectively. Considering that connectivity is both hypo- and hyper-, a combination of burst and noise may be essential to normalize dysconnectivity in MDD, with burst stimulation potentially increasing connectivity and noise stimulation desynchronizing activity, i.e., decreasing connectivity.

Yet still another form of neurostimulation can be developed, called reconditioning stimulation. The concept is that electrical stimulation is paired to external stimuli, as first proposed in a seminal paper in tinnitus. This concept can be adjusted to treating depression and has as advantage that the paired stimulation exerts a learning effect on the brain, instead of only suppressing hyperactivity or blocking hyperconnectivity, which is the mainstay of current neurostimulation approaches. The adaptation would be to pair external hedonic stimuli to rewarding stimuli delivered at different parts of the reward circuitry.

In summary, novel targets combined with novel stimulation designs pave the way for improved treatments for MDD and entirely novel approaches, such as reconditioning stimulation, might be yet another approach to treat this most debilitating of brain disorders.

### Pathway-Specific Targeting for Subcallosal Cingulate Deep Brain Stimulation

DBS of the subcallosal cingulate white matter (SCC DBS) is an emerging strategy for treatment-resistant depression (56). Clinical trials show response rates at 6 months across studies range from 41 to 66% with sustained and increased response over time (40, 73). A challenge to effectively disseminate this nascent treatment remains in the fact that there is an absence of biomarkers to guide lead placement or to titrate stimulation parameters during follow-up care. Unlike PD, where intraoperative electrophysiology is routinely employed to define the anatomical–functional placement of the lead and titrate stimulus parameters to moderate symptoms in real time, such mechanistically guided biomarkers for depression are lacking. Furthermore, the SCC target is in the white matter, without demarcated anatomical boundaries. As such, individualized mapping of the target and its precise cortical connections is a critical first step to standardize the procedure.

Targeting the SCC white matter was based on converging imaging data demonstrating changes in SCC activity with antidepressant response to a variety of standard treatments (74, 75). Selection of this target was further supported by an extensive literature demonstrating monosynaptic connections between the subcallosal cingulate and specific frontal, limbic, subcortical, and brain stem sites involved in mood regulation, depression, and the antidepressant response (76, 77). Specific placement of the DBS electrodes was therefore determined by local anatomy. Approximate coordinates were derived from PET imaging studies localizing the subcallosal cingulate region (Brodmann area 25) and adjacent white matter and were then combined with anatomical landmarks identified in standard neurosurgical atlases. Tractography-guided connectomic approach to SCC electrode implantation has been found to improve the precision of surgical targeting following an initial feasibility study (49, 78).

**Figure 6:**
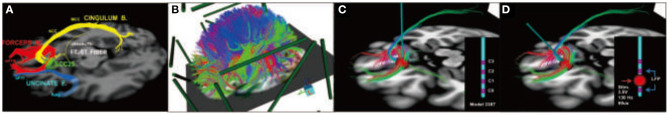
Prospective targeting of these four white matter bundles can be performed reliably in individual patients, and use of this method improves long-term outcomes. **(A)** Four-bundle white matter “blueprint”: cingulum (yellow), uncinate fasciculus (blue), forceps minor (red), frontal-striatal (white). **(B)** Whole-brain tractography loaded in patient-specific stereotactic frame space using StimVision. **(C)** Visualizing tracts passing through the volume of tissue activated (VTA) to define optimal target location that best visually matched the “blueprint.” **(D)** Finalization of lead trajectory with the neurosurgeon to avoid cerebral vasculature and choosing the point of entry.

In the latest developments by Choi et al. (79) refinement of surgical targeting has been aided by using tractography guidance. White matter pathways have now been mapped in responders and non-responders in the first study cohort to define the necessary and sufficient pathways that must be stimulated to achieve a full antidepressant effect (78). These maps utilize individualized models of the volume of tissue activated (VTA) derived from each patient's diffusion tractography scan (80). Successful prospective targeting in the most recent cases of 11 patients has resulted in successful mapping of responders group. These latest results confirm that prospective targeting of four key white matter bundles (cingulum, uncinate fasciculus, forceps minor, and frontal-striatal; Figure 6) can be performed reliably in individual patients, and use of this method improves long-term outcomes.

### Perspectives of Deep Brain Stimulation for Memory Disorders

Memory deficits are a characteristic feature of numerous neuropsychiatric disorders, including various forms of dementias. To date, no effective treatment exists for memory deficits in dementia. Commonly used medications are acetylcholinesterase inhibitors, but the overall response is not very satisfactory (81). Neuromodulation strategies, including DBS, have been recently proposed for the treatment of these conditions.

The use of electrical stimulation for the study of memory problems is not new. In animal models, stimulation of various brain regions has been conducted in attempts to investigate physiological aspects in several learning and memory paradigms (82, 83). Early reports administering high current intensities to limbic structures in rodents undergoing memory tests reported stimulation-induced memory deterioration (82, 84). In contrast, stimulation regimens suited to induce plasticity (85, 86) were found to improve memory.

In humans, memory improvement has been reported in patients with epilepsy receiving entorhinal cortex (EC) (87) or anterior nucleus of thalamus (ANT) DBS (88). However, impairment has also been described particularly when stimulation was delivered acutely at relatively high currents (89). A patient with morbid obesity treated with DBS in the hypothalamic/forniceal region presented dejà vu sensations (90). In this same patient, stimulation was found to modulate the activity of the mesial temporal lobe and improve hippocampal memory function, as measured with neuropsychological testing (90).

A few years ago, a phase 1 clinical trial was conducted to test the safety of fornix DBS in six patients with AD (91). In addition to promising clinical findings, DBS was shown to modulate the activity of mesial temporal lobe structures and increase brain metabolism in temporal and parietal regions, as revealed by positron emission tomography (PET) scan (91). Following that study, a phase II trial of fornix DBS in mild AD (ADVANCE) was conducted (92). Forty-two patients were recruited in centers across the United States and Canada. It consisted of a 12-month double-blinded randomized controlled study comparing active and sham stimulation. When all patients were considered, no significant differences were observed in the Alzheimer's Disease Assessment Scale–Cognitive (ADASCog) 13 scores between groups (92). Interestingly, however, patients older than 65 receiving DBS seemed to have had a slower disease progression (though no significant differences were detected compared to sham-treated individuals of the same age group) (92). Another DBS target proposed for the treatment of AD is the nucleus basalis of Meynert, with preliminary studies showing promising results in different clinical types (93).

To explain potential mechanisms of stimulation, preclinical work has been conducted. In one of these studies, Gratwicke et al. (94, 95) stimulated the EC of AD transgenic animals. The authors found significant improvements in animals receiving DBS compared to sham treatment in the Morris water maze and novel object recognition tests. Remarkably, DBS reduced the number of Aβ plaques, as well as tau, phosphorylated tau, and amyloid precursor protein (APP) in the hippocampus of transgenic animals (94).

Taken together, the effects of stimulation on memory seem to vary as a function of the current intensity, target, and duration of treatment. Promising results have been reported in degenerative disorders, including AD. In animal models, DBS has been shown not only to improve memory function but also to have neuroprotective effects. Recent clinical trials in AD patients have shown promising enough results to warrant large-scale studies.

## Latest Approaches in Deep Brain Stimulation for Movement Disorders

### From Primate to Man: Pedunculopontine Nucleus Stimulation as a Therapy for Patients With Parkinsonian Disorders

FOG and falls are two of the most disabling symptoms of PD affecting upward of 10% of such patients (96). The introduction of L-DOPA in 1975 was so miraculous in reversing the cardinal signs of PD that it led to an initial discontinuation of functional neurosurgery, as it was felt that a single drug could be found to have similar beneficial effects with less potential side effects in movement disorders (97). However, it became apparent that with time, after 5 years, upward of 60% of patients would develop crippling medication-induced side effects such as dyskinesias. To move forward, a better understanding of the disease was needed. The problem was that, at that time, there was no animal model to better understand the disease pathology.

Serendipity came into play with the report in 1983 by Langston and Ballard (98) and Langston et al. (99) of a young man admitted in a presumed catatonic state unresponsive to psychiatric therapies. However, when given L-DOPA, his symptoms were reversed. Subsequently, a series of these patients were reported to have been rendered parkinsonian by self-administration of a pethidine analog, methyl-phenyl-tetrahydropyridine (MPTP). After one patient overdosed on cocaine and died, the autopsy conducted showed the changes in the brain similar to those seen in PD, particularly loss of nigral dopaminergic neurons. In 1983, Burns et al. (100) administered MPTP to NHPs and produced an experimental model of PD, mimicking bradykinesia, forward flexed posture, and rigidity. These animals were also highly responsive to L-DOPA therapy.

Subsequent studies using the MPTP-lesioned NHP model of PD with electrophysiology and 2-deoxyglucose (2-DG) studies (101) led to a key pathophyisiological understanding of PD, in which loss of nigral dopamine leads to disinhibition of the STN, causing an excessive inhibitory drive from the medial pallidum to ascending and descending pathways to the thalamus and upper brain stem. Two pioneering studies confirmed that lesioning the STN, using either neurotoxin (102) or surgical radiofrequency electrodes (103), reversed experimental parkinsonism. Lesioning the STN was not considered an option for fear of inducing hemiballism; therefore, it was the finding that high-frequency stimulation of the STN in the NHP model that made it clinically applicable. Very soon after, STN HFS became to be the most accepted treatment for advanced PD.

With time, it became apparent that even with medication and STN-DBS, PD patients were not resistant to FOG and falls. Certain lines of evidence suggest that the upper brain stem might be relevant to understanding this. For example, in decerebrate rats, cats, and dogs, electrical or chemical stimulation in the mesencephalic motor region induces walking. The particular brain region, the pedunculopontine nucleus (PPN), was shown to degenerate in PD and other akinetic disorders such as multiple system atrophy (MSA) and progressive supranuclear palsy (PSP). In addition, 2-DG studies have indicated that MPTP-lesioned NHPs have excessive inhibition of the PPN.

A lesion or high-frequency stimulation of the PPN in healthy NHPs induces akinesia (104). Once rendered parkinsonian with MPTP administration, microinjections of bicuculline [gamma aminobutyric acid (GABA) antagonist] directly into the PPN reverses akinesia and imbalance, as does low-frequency PPN stimulation (105). The finding that PPN stimulation alleviated movement abnormalities in the MPTP-lesioned NHP was translated rapidly to treat PD patients by clinical groups in the UK (Bristol) and Italy (Rome), with low-frequency stimulation (around 20–30 Hz) being employed (106, 107). These early clinical studies noted an effect of PPN stimulation that mimicked those found in the NHPs, which was an improvement in akinesia, in addition to gait and posture. This was later followed by a clinical study of six PD patients with dual STN and PPN stimulation (108). Results suggested modest improvements in akinesia and more marked beneficial effects in FOG and postural instability, with the suggestion of STN and PPN DBS being complementary. However, a major issue arose regarding the target that appeared to lie in the neighboring peri-peduncular nucleus. This has since prompted a debate on the exact location of the PPN, and this remains a controversial issue (109–112).

Two further clinical series have reported PPN stimulation in two differing scenarios (1) dual bilateral STN and PPN stimulation and (2) single-target unilateral PPN stimulation (113, 114). These studies have reported very modest therapeutic effects of PPN stimulation, largely limited to FOG and postural instability. One study found very limited effects even on FOG and questioned the clinical utility of this treatment (113). However, several aspects in the clinical application of PPN stimulation in these studies could have affected therapeutic efficacy. Indeed, some patients were selected for PPN stimulation with severe motor fluctuations requiring STN stimulation and variable degrees of gait disturbance (108, 113). For example, PD patients were selected for PPN stimulation to treat FOG that developed during STN stimulation. Other patients had been selected for PPN stimulation who had not experienced FOG that persisted “on medication” or having recurrent falls (113, 114). It remains possible that co-stimulation of the STN could influence the efficacy of PPN stimulation due to the substantial reciprocal connections between the two targets (115). In this regard, it should be noted that high-frequency stimulation required for STN stimulation (i.e., 130 Hz) appears to worsen gait when delivered to the PPN. It has been found that the PPN was targeted above the pontomesencephalic junction with choline-acetyltransferase 5 (ChAT5) staining studies in humans, suggesting that lead placement could have missed the caudal extent of the nucleus, which is most degenerate in PD (116–118). Moreover, a clinical study by Thevathasan et al. (119) demonstrated that bilateral PPN stimulation provides a greater therapeutic effect over unilateral stimulation.

It is apparent that there are some questions remaining regarding the effect of PPN stimulation in parkinsonian FOG and falls, i.e., the exact target and the best patient candidates are still debated. It is noteworthy that the outcomes measured with UPDRS may lack sensitivity for gait and posture. Indeed, the precise effects of PPN stimulation on motor function in PD including gait are not established (120). However, in recent years, it has been reported in a meta-analysis of several well-documented PPN stimulation studies that patients ON or OFF medication are better with PPN stimulation (121).

### Novel Neuromodulation Applications for Gait Disorders in Parkinson's Disease

Postural instability and gait disorders (PIGDs) are debilitating phenomena that frequently impair locomotion and can significantly affect quality of life in PD patients (122). Prevalence of PIGD tends to follow the severity of disease; it is the most common cause of falls, which are associated with an increase in morbidity and mortality in PD (122). Besides the effects of STN and GPi DBS on PIGD responsive to levodopa, PPN DBS was the first neuromodulatory technique directly applied for the treatment of PIGD, with success recorded in many reports (123). In addition, there have been some reports on dual stimulation STN/SNr and VFS of STN as possible neuromodulatory methods for PIGD (124, 125).

Since the first experimental report from Fuentes et al. (126) showing that spinal cord stimulation (SCS) could enhance locomotion in murine PD models, and more recently in NHPs (127), SCS has been considered as a possible treatment for FOG in PD. Increasing evidence suggests that SCS improves treatment-resistant PIGD in PD patients (128). Recently, Pinto de Souza et al. (129) have reported positive effects of high-frequency SCS (300 Hz) on gait, improving the performance in various gait tests, which were reproduced during double-blinded assessments. It was also seen that continuous SCS chronically reduced FOG episodes, improved UPDRS-III motor scale scores, and self-reported quality of life (129). These results are in line with previous clinical observations and findings recorded in parkinsonian animal models (126, 127). More recently, Samotus et al. (130) also showed positive effects of SCS for gait dysfunction in PD patients. In comparison to early reports, the selection of PD patients with locomotor problems were more precise (130), in which motor symptoms, gait performance, and FOG were closely followed with adequate evaluations.

Despite that positive results have been reported, the mechanisms by which SCS may improve FOG are still elusive. Considering that the exact mechanism of FOG itself is also not completely understood, the study of SCS might converge with the study of FOG. Normal gait requires an exact coordination of postural adjustment in advance of each step forward, namely, anticipatory postural adjustment (APA). During imminent FOG episodes, the intention to walk is uncoupled from the triggering of APA, with consequent failure of the forward movement. This often results in knee trembling and failure to initiate gait. In PD, FOG episodes are associated with deficient APA. Physiological evidence, functional imaging, and clinicopathologic studies suggest that FOG is mainly associated with disorders of frontal cortical regions [e.g., supplementary motor area (SMA)] that comprise a known brain circuitry dedicated to APA control (131). In NHPs, SCS increases neuronal firing of the primary motor cortex and decreases pathological cortico-striatal synchronous low-frequency waves showing that SCS does influence the oscillatory activity in multiple structures of brain motor circuits (127). In fact, SCS may disrupt the aberrant inhibition from the GPi to the thalamus and SMA. As part of the circuit that controls APA, the SMA has corticofugal projections to the PPN, a region particularly involved in gait initiation (as described above). Since the activity of SMA, globus pallidus, and PPN is abnormal in PD patients with FOG, SCS could potentially modulate this circuit and improve APA and gait initiation.

**Figure 7:**
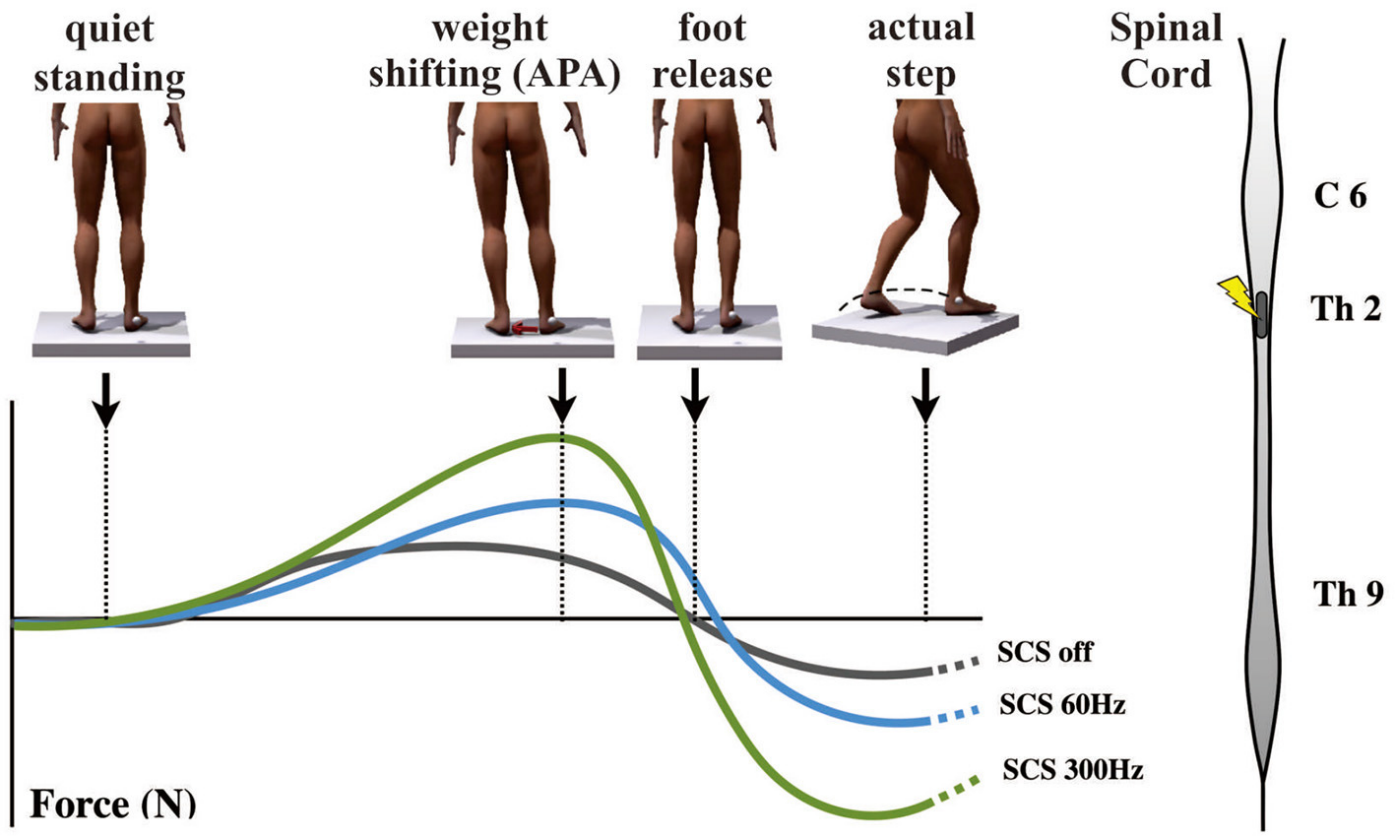
Effects of spinal cord stimulation on postural control in Parkinson's disease patients with freezing of gait (132). On the top left, the figures show the representation of the step initiation task from quiet standing to the actual step, showing the marker on the right malleolus to detect the moment that the foot clears the floor. The graph below shows body weight shifting toward the supporting leg [anticipatory postural adjustment (APA)] during the three different conditions [gray curve, spinal cord stimulation (SCS) OFF; blue curve, 60 Hz-CS; green curve, 300 Hz-SCS]. On the right is a representation of the spinal cord showing the site of stimulation (T2) used in the same report.

Recent work by de Lima-Pardini et al. (132) has recently reported that SCS at 300 Hz effectively reduces the time of FOG with simultaneous correction of altered APA, which is reported in PD patients with FOG (Figure 7). These results corroborated with initial clinical data demonstrating significant progress toward revealing mechanisms by which SCS may improve FOG. It is possible that by stimulating ascending spinal pathways, SCS may correct pathological oscillatory activity in the circuits that mediate FOG, subsequently inhibiting episodes of FOG in PD patients. Conversely, SCS has failed to improve reactive posture control. It is possible that SCS may have different effects on the two mechanisms of postural control that are known to be reactive and anticipatory. While APA mechanisms are thought to be dependent on thalamo-cortical-striatal loops highly influenced by attentional and environmental changes, reactive posture control to external and unpredictable triggers relies on neuronal circuitries involving the brain stem and spinal cord with less influence from the cortex.

Despite various reports on the positive effects of SCS on FOG, there is still skepticism regarding this treatment since some PD symptoms can improve remarkably with placebo or during startle responses upon threats. In order to differentiate the effect of SCS from placebo, Pinto de Souza et al. (129) performed a double-blind study comparing the effects of stimulation at 300 and 60 Hz, once both frequencies elicited indistinguishable paresthesia. It was found that only the stimulation at 300 Hz improved gait performance and reduced episodes of FOG, while the effects of the lower frequency were similar to no stimulation.

In summary, there is increasing evidence for SCS-induced improvements in gait disturbances for PD, especially FOG. However, evidence from comparative studies with larger patient populations and data from prospective placebo-controlled trials is still lacking. The exact mechanisms and circuits mediating the expression of FOG are still uncertain. In addition, the optimal level for spinal stimulation, specific structures (segmental short circuits or ascending tracts) for effects of SCS, the most effective electrode geometry and specific parameters of stimulation remain undefined (133). Besides its potential therapeutic use, the development of SCS for the treatment of PD symptoms may also contribute to a better understanding of locomotor behaviors and complex pathophysiology of neurological disturbances, specifically FOG (134).

### Deep Brain Stimulation to Enhance Chronic Post-stroke Rehabilitation

Ischemic stroke is a major cause of long-term disability in the industrialized world, with chronic debilitating motor impairments significantly impacting quality of life for more than one third of stroke survivors. Current standard-of-care treatment for those left with motor sequelae is largely limited to subacute physical therapy. However, long-term disabling deficits for most patients persist despite best efforts. As a result, there is substantial interest in identifying new ways to enhance post-stroke recovery and rehabilitation, including invasive and non-invasive neurostimulation-based approaches. Progress has been limited to date, however, with most clinical studies yielding limited or variable efficacy in improving motor function using current approaches.

**Figure 8:**
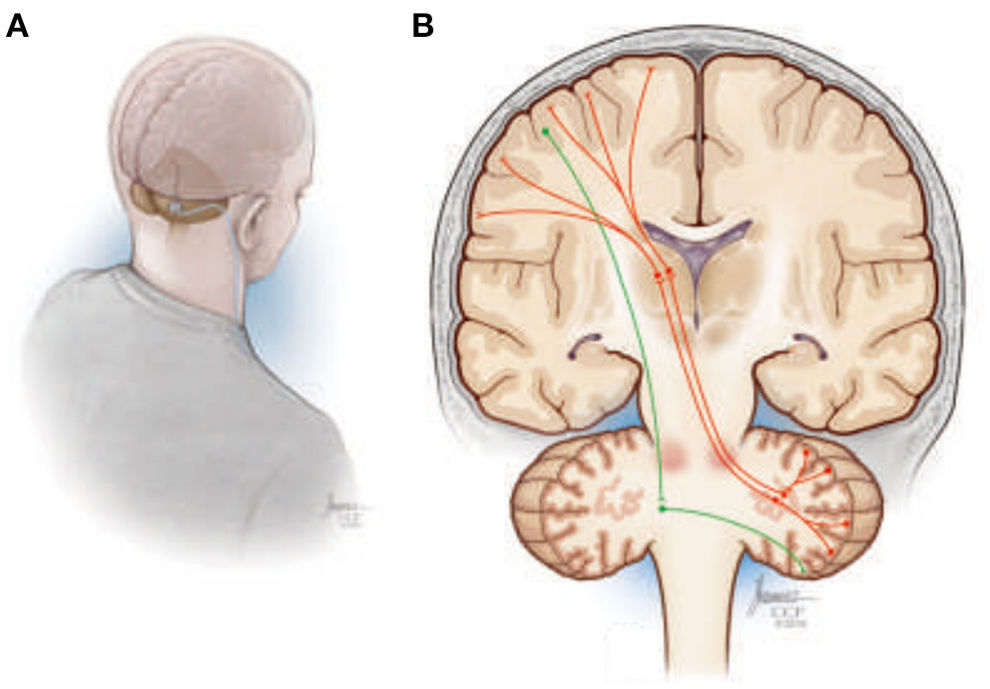
**(A)** Illustration depicting a suboccipital approach to delivering deep brain stimulation therapy to the cerebellar dentate nucleus. **(B)** Simplified overview of the human dentatothalamocortical (DTC) and corticopontocerebellar (CPC) pathways. The DTC (red) projects through the ipsilateral superior cerebellar peduncle, decussating at the level of the pons, to terminate in contralateral thalamus, where its activity influences widespread thalamocortical interactions. The CPC is shown in green descending from the cortex, decussating in the ipsilateral pons, and terminating in the contralateral cerebellar hemisphere.

Machado and Baker (135) have previously proposed that chronic stimulation of cerebellar dentate nucleus (DN; Figure 8A) should, through activation of the net excitatory glutamatergic dentatothalamocortical pathway, upregulate thalamocortical activity and cerebral cortical excitability across prefrontal, frontal, and parietal cortical regions (Figure 8B), establishing a basal environment more compatible with functional neuroplastic reorganization. The work over the past decade, using preclinical models of middle cerebral artery ischemia, that stimulation of the lateral cerebellar nucleus (the rodent homolog of the human DN) does indeed facilitate motor recovery when paired with rehabilitation, with the magnitude of the effect sensitive to stimulation frequency (136, 137). Moreover, the electrophysiological and histological data implicate frequency-specific changes in cortical excitability and enhanced functional reorganization of surviving perilesional cortex as potential therapeutic mechanisms, with improvements in motor function accompanied by increased expression of markers of synaptic plasticity, synaptogenesis, and neurogenesis in the perilesional cortex (138, 139).

Based upon the preclinical work, a first-in-human trial (NCT02835443) to translate DN-DBS as a treatment for upper extremity hemiparesis in chronic post-stroke patients commenced in 2016. With additional support from the National Institutes of Health Brain Initiative, the trial will extend beyond establishing the safety, feasibility, and efficacy of the approach to directly examine the acute and chronic effects of DN-DBS on cerebral cortical excitability and motor representation using TMS-based techniques as well as the topography of motor-related LFP activity in the DN region. All these data are further being incorporated into MRI-based patient-specific anatomical models of the deep cerebellar region to facilitate next-generation lead design and targeting techniques, while using the LFP data to develop physiological classifiers to inform future treatment paradigms, including possible closed-loop approaches. Finally, ongoing preclinical studies are focusing on therapy refinement and optimization, including a potential role for a more physiologic-based, closed-loop stimulation system that more directly stimulates delivery with motor activity.

### Current Clinical Deep Brain Stimulation Approaches for Dystonia

DBS of the GPi is an effective treatment for medical refractory dystonia and reduces not only motor impairment but also other disabilities (140–144). Within the last two decades, DBS has progressively evolved into a widely available therapeutic strategy for generalized and segmental dystonia. More recently, it was also successfully applied in patients with cervical dystonia or cases that were resistant to botulinum toxin treatment (145). Long-term effects and side effects of this potentially lifelong therapy are of special interest. More recent retrospective reports of DBS for dystonia includes follow-up periods of 5–7 years (146–149) with sometimes even longer observation periods in individual cases, i.e., 6–10 years (148) and overall good clinical outcome of 50–80% mean improvement of dystonia. However, individual factors that remain reliable for predicting DBS outcome in dystonia are still difficult to define. Several factors such as gene mutation status, age at surgery, disease duration, presence of musculoskeletal deformities, predominance of phasic vs. tonic movements, the size of the globus pallidus, and optimal stimulation parameters are widely discussed to have a possible influence on stimulation effects (148–152). Brüggemann et al. (150) now report that patients genetically confirmed with DYT1 and DYT6 dystonia have significant and enduring effects of pallidal stimulation. Furthermore, Isaias et al. (149) and Lumsden et al. (151) report cases with disease duration being an important factor, e.g., with respect to developing fixed musculoskeletal deformities, also highlighting the importance to differentiate between isolated dystonia and patients with combined/complex dystonia because the latter shows a significantly reduced benefit of pallidal DBS.

While the clinical benefit of DBS in cervical and other focal dystonias is well-documented, the underlying therapeutic mechanism remains to be elucidated. Converging evidence points to a modulation of aberrant neural population activity in the basal ganglia through high-frequency stimulation (9, 153, 154). In recent years, DBS has enabled the unique opportunity to record oscillatory activity as LFPs directly from the basal ganglia during surgery and in a postoperative interval, with the DBS electrodes externalized. Here, oscillatory patterns of pallidal LFPs were found to differ in a disease-specific manner (155, 156). The best characterized pathological oscillatory phenomenon has been described in patients with PD, where STN β oscillatory activity (13–30 Hz) at rest is suppressed by dopaminergic medication and is directly correlated with patient symptom severity (9). In dystonia, low-frequency activity in the θ-α range (4–12 Hz; subsequently referred to as θ, as most peaks in dystonia are in the 4–10-Hz range) is predominant in the GPi and correlates with symptom severity (157). Indeed, θ activity in dystonia patients with phasic movements has been shown to be suppressed by high-frequency DBS (154). Thus, pallidal θ activity has been proposed as a potential pathophysiological hallmark of dystonia. It can be envisioned that adaptive closed-loop DBS using pallidal θ activity as a biomarker could be efficiently used for controlling dystonic motor symptoms in patients.

### Automatic Classification of Pallidal Borders During Awake and Asleep Deep Brain Stimulation Procedures for Dystonia

DBS of the GPi in patients with dystonia can reduce motor symptoms and improve their quality of life (158–160). With the current limits of today's brain imaging techniques in resolution, distortion, and possible brain shift (161), together with the broad distribution of DBS centers (>1,000 worldwide with many non-academic centers), the outcome of many DBS procedures might be less optimal because of mis-localization of the DBS leads (13, 162). To enable better localization of the DBS target, pallidal borders can be visibly and audibly detected by electrophysiological microelectrode recordings (MERs) during DBS procedures. Even given ideal conditions, the detection of the striato-pallidal borders is never an easy task even for an expert electrophysiologist.

Previously, it has reported a real-time automated procedure (163, 164) for the detection of the borders and subdomains of the STN using hidden Markov models (HMMs) in PD patients (165). Bergman et al. also reported an algorithm for detection of the striato-pallidal borders, with a dataset including 116 GPi trajectories from 42 patients consisting of 11,774 MERs in five classes of disease (awake PD patients, awake and lightly anesthetized genetic and non-genetic dystonia patients (166), with the current work now under review for publication; *Journal of Neuro-engineering*). Using the L_1_-distance measure in root mean square (RMS) and power spectral densities of the MER, Bergman et al. has found that awake and light anesthesia (with sevoflurane and N_2_O, minimum alveolar concentration (MAC) = 0.3–0.6) dystonia classes with and without anesthesia can be merged. Therefore, depth (MAC) of anesthesia was reduced 10–15 min before the beginning of the MER and restored deep surgical anesthesia after the end of the MER exploration in each hemisphere. It was found that significant differences exist between the RMS and spectral features of the striato-pallidal trajectory. Bergman et al. reported training on the HMM on trajectories with striato-pallidal labels as inputs in three different disease classes (PD, genetic and non-genetic dystonia) using the decision of an expert electrophysiologist as gold standard labels. Then, the performance of the HMM algorithm was tested with a leave-one-out cross-validation. The HMM was found to achieve performance on par with an expert electrophysiologist across the striatum-GPe, GPe-GPi, and GPi-exit transitions in the three disease classes (167).

In conclusion, as for STN DBS, GPi automated navigation systems can potentially shorten the length of electrophysiological mapping to <15 min per hemisphere, while implanting the DBS lead within the optimal location. A reduced procedure time and improved targeting would be expected to lead to better clinical outcomes in GPi DBS therapy for dystonia.

### Long-Term Awake Multi-Electrode Monitoring in Children With Acquired Combined Dystonia

Acquired combined dystonia remains a difficult disorder to treat partly because of the variability of causes and symptoms. Dystonia has multiple potential anatomic origins, including basal ganglia, cerebellum, thalamus, and prefrontal cortex. Despite these origins, the specific postures generated are often remarkably similar, and a review of 3 years of clinic videos shows that almost all children have at least one of seven stereotypical postures, no matter what their underlying etiology (168). This has led to the conjecture that these postures are due to similar somatotopy within the motor cortex, so that any brain region capable of stimulating contiguous regions of motor cortex will cause a similar posture. This finding is reminiscent of the very limited sets of postures seen with long-train electrical stimulation of the motor cortex (169). If unfocused stimulation of motor cortex is the final common pathway, then dystonia is a very non-specific symptom, and successful treatment using DBS will require uncovering the anatomic origin of the disorder in each child.

**Figure 9:**
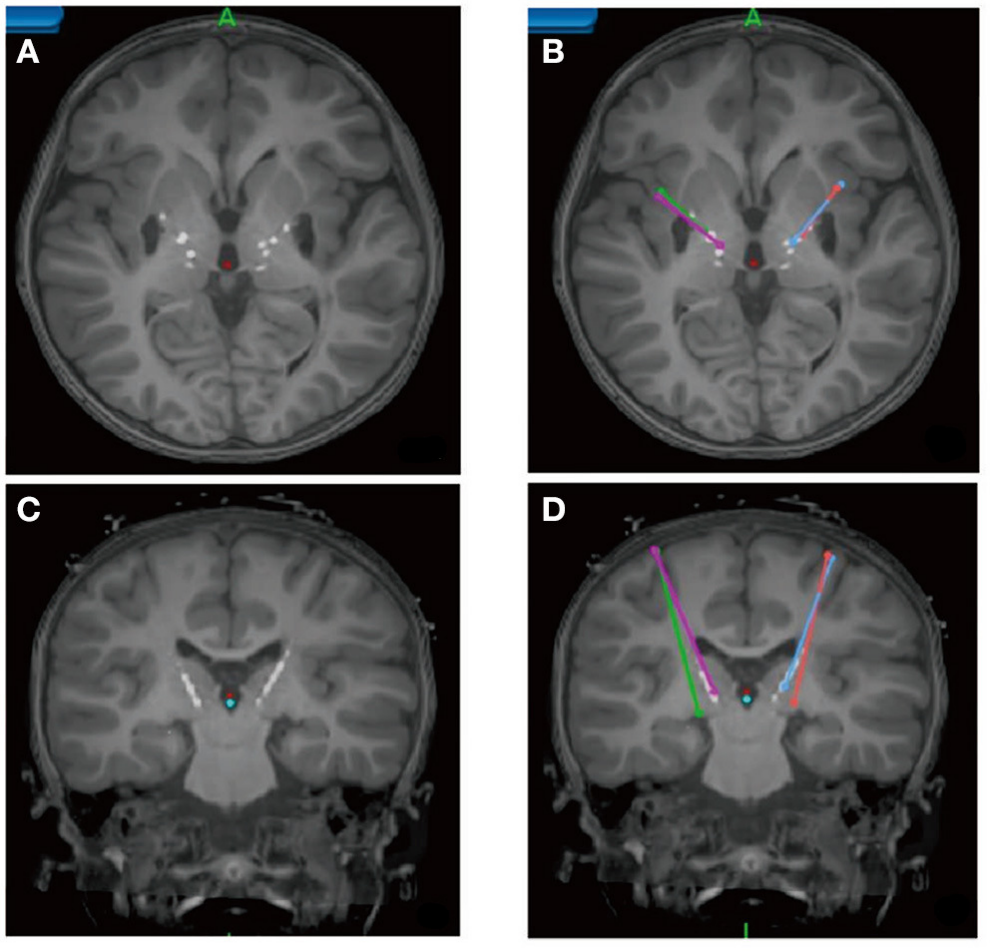
Pediatric Deep Brain Stimulation Using Awake Recording and Stimulation for Target Selection in an Inpatient Neuromodulation Monitoring Unit (170). Axial **(A,B)** and coronal **(C,D)** MRI showing the position of temporary leads within the basal ganglia and thalamus. Stereotactic planning trajectories are shown in **(B,D)**.

In order to do this, Sanger et al. (170) have developed a new procedure that includes test stimulation and recording from multiple externalized electrodes while children are admitted to a neuromodulation monitoring unit (NMU). Up to 10 electrodes (Figure 9), each with 16 contacts, are implanted in multiple regions of the basal ganglia and thalamus, targeting the most likely pathways for uncontrolled cortical stimulation. Test stimulation can be used to determine efficacy and any side effects of stimulation of each region. This is particularly helpful in the thalamus, for which the effects of stimulation are usually immediate. In the pallidum, effects of stimulation may require weeks or months to determine; therefore, test stimulation is primarily used to find regions that are free of adverse effects that might limit flexibility of stimulation.

Recording yields single-unit activity that can be correlated against surface electromyography (EMG) to determine regions that are more or less likely to be carrying dystonic signals. In particular, a region that does not change activity during dystonic contractions is unlikely to be a mediator of dystonia and unlikely to be a candidate target for DBS. Sanger et al. (170) have recorded so far from 20 children with acquired combined dystonia. Contrary to data from adults, in NHPs and children with primary dystonia, the group have found that baseline activity in the pallidum is low and increases with dystonic muscle contractions. In fact, all regions activate with dystonic spasms, including pallidum and multiple thalamic subnuclei (VIM, VPL, Vo, and VA). Although the pallidum inhibits the thalamus, activity in the pallidum is positively correlated with activity in the thalamus in all children. This suggests a loss of the normal inhibition and loss of specificity of activity within these regions. Overflow to contralateral muscles is evident within cerebellar projection pathways, and dystonic activity is always much higher and less focused than voluntary activity. Taken together, the results reported here suggest a generalized lack of specificity and both reversal of normal activity and hyperexcitability throughout the basal ganglia/thalamus circuit. Since the output of this circuit projects to motor cortex, these findings are consistent with the hypothesis of non-specific cortical drive as the mediator of dystonic postures.

Use of the NMU procedure by Sanger et al. (170) has allowed for finding more precise patient-specific targets for children. The group always implants four leads: usually two within the GPi and two within the optimal region of the thalamus for each child. Subsequent programming suggests that GPi DBS is the most effective for hypertonic components of dystonia, whereas thalamic DBS is the most effective for the hyperkinetic components of dystonia. Preliminary analysis of outcomes data shows significantly improved outcomes using the new procedure and four-lead DBS. This is a promising new procedure that will yield both improved outcomes for children with acquired combined dystonia, as well as detailed knowledge on the physiological mechanisms underlying this disabling condition.

## Conclusions

This report summarizes the information presented in the first DBS initiative meeting held at the NELN of Tsinghua University. The collective group addressed foreseeable challenges in DBS therapy and recent clinical approaches with technological advancements. In-depth discussions were held on the connectome approach in DBS, novel developments in 3T MRI-compatible DBS devices and neural recording technologies for understanding disease pathophysiology, and pursuing new clinical approaches and indications using such advancements. This meeting marks a unique milestone in developing global DBS research using state-of-the-art technologies for rapid clinical translation.

## Author Contributions

All authors listed have made a substantial, direct and intellectual contribution to the work, and approved it for publication.

## Conflict of Interest

The authors declare that the research was conducted in the absence of any commercial or financial relationships that could be construed as a potential conflict of interest.

## Abbreviations

2-DG: 2-deoxyglucose
3T MRI: 3-Tesla magnetic resonance imaging
AD: Alzheimer's disease
AI: artificial intelligence
ALIC: anterior limb of the internal capsule
ANT: anterior nucleus of thalamus
APA: anticipatory postural adjustment
APP: amyloid precursor protein
ChAT5: choline-acetyltransferase 5
dACC: dorsal anterior cingulate cortex
DBS: deep brain stimulation
dMRI: diffusion magnetic resonance imaging
DN: dentate nucleus
EC: entorhinal cortex
FOG: freezing of gait
GPi: globus pallidus internal segment
HMMs: hidden Markov models
IEC: International Electrotechnical Commission
IPG: implantable pulse generator
LFPs: local field potentials
MDD: major depressive disorder
MERs: microelectrode recordings
MFB: medial forebrain bundle
MPTP: methyl-phenyl-tetrahydropyridine
MSA: multiple system atrophy
NELN: National Engineering Laboratory for Neuromodulation
NHP: nonhuman primate
NMU: neuromodulation monitoring unit
OCD: obsessive–compulsive disorder
OFC: orbitofrontal cortex
PD: Parkinson's disease
PET: positron emission tomography
PIGDs: postural instability and gait disorders
PPN: pedunculopontine nucleus
PSP: progressive supranuclear palsy
RF: radiofrequency
RMS: root mean square
SAR: specific absorption rate
SCC: subgenual cingulate cortex
SCGwm: subgenual cingulate gyrus white matter
SCS: spinal cord stimulation
sl-MFB: superolateral branch of the medial forebrain bundle
STN: subthalamic nucleus
TMS: transcranial magnetic stimulation
TUG: Timed Up and Go
VC/VS: ventral capsule/ventral striatum
VIM: ventral intermediate
vmPFC: ventromedial prefrontal cortex
VFS: variable frequency stimulation
VTA: volume of tissue activated.
